# The Utilization of Space by Juvenile Spotted Seals (*Phoca largha*) from Peter the Great Bay (East Sea/Sea of Japan) Throughout the Year

**DOI:** 10.3390/ani16172676

**Published:** 2026-08-26

**Authors:** Peter Alekseevich Permyakov, Sergey Dmitrievich Ryazanov, Alexey Mikhailovich Trukhin, Vyacheslav Borisovich Lobanov, Hyun Woo Kim, Sora Kim

**Affiliations:** 1 V.I. Il’ichev Pacific Oceanological Institute, Far Eastern Branch, Russian Academy of Sciences, Vladivostok 690041, Russia; ryazanov@poi.dvo.ru (S.D.R.); trukhin@poi.dvo.ru (A.M.T.); lobanov@poi.dvo.ru (V.B.L.); 2 Coastal Water Fisheries Resources Research Division, National Institute of Fisheries Science, Busan 46083, Republic of Korea; 3 Cetacean Research Institute, National Institute of Fisheries Science, Ulsan 44780, Republic of Korea; sora1012@korea.kr

**Keywords:** spotted seal (*Phoca largha*), juvenile individuals, summer migrations, East Sea/Sea of Japan

## Abstract

Spotted seals (*Phoca largha*) exhibit an unusual ecological feature within the population of Peter the Great Bay (PGB). Following the breeding and molting seasons, one group performs long-distance foraging migrations, whereas another group remains in the bay year-round. To examine how space use differs between these two groups, nine satellite tags were deployed on juvenile spotted seals within PGB between 2017 and 2022: four seals were tagged at the rookery located on the Rimsky-Korsakov Archipelago (RKA), whereas the remaining five were tagged at the haul-out on the Verkhovskogo Islands (VI). Spotted seals tagged at RKA undertook long summer migrations toward the northeast of the East Sea/Sea of Japan and subsequently to the south of the Okhotsk Sea. Four spotted seals tagged at VI remained in PGB throughout the warm season. Seals were allocated to two clusters based on median speed during transit travel. The slower-moving group consisted of three spotted seals that remained in PGB; these seals also made fewer transit passages and used smaller areas of water.

## Simple Summary

Spotted seals (*Phoca largha*) in Peter the Great Bay (East Sea/Sea of Japan) exhibit an atypical ecological segregation: during the warm season, the majority of the population migrates long distances outside the bay for foraging, whereas a smaller portion remains within the bay year-round. Satellite tags were deployed on nine juvenile spotted seals in the bay—four at the local rookery and five at distal haul-outs. Seals tagged at the rookery performed long-distance foraging migrations, whereas those tagged at distal haul-outs generally did not. The three nonmigrating spotted seals moved slowly and used relatively small areas of water. Conversely, the migrating spotted seals showed elevated activity levels and, evidently, greater energetic demands. This study is the first to show that seals breeding at the same location can exhibit markedly different patterns of space use.

## 1. Introduction

Degradation of ice coverage currently observed in high latitudes due to global warming causes a deterioration of habitats, usable by fast-ice-dependant marine mammals. In this connection population-specific adaptations to variable sea ice regimes remain an important topic in marine mammal behavioral ecology. Certain populations of pagophilic marine mammals, whose centers of reproduction occur in lower latitudes, can represent a particular scientific interest, as their adaptations develop historically under least-stressful conditions. One such population—a rare non-fast ice breeding colony of spotted seal (*Phoca largha* Pallas, 1811)—exists in Peter the Great Bay (hereinafter: PGB), in the northwest of the East Sea/Sea of Japan (hereinafter: ES/SJ). This population can serve as a natural study system for exploration of life history divergence.

The spotted seal is an abundant species of Phocid seals (*Phocidae* Gray, 1825) that inhabits near-shore waters in the Northern Pacific Ocean and the Pacific sector of the Arctic [1]. Eight isolated reproductive centers of spotted seals are recognized within its range: three in the Bering Sea, two in the Okhotsk Sea (hereinafter OS), two in the ES/SJ, and one in the Yellow Sea [2,3]. Throughout most of its range, the spotted seal leads a pagophilic way of life, breeding and nursing its offspring on floating ice. However, the population inhabiting Peter the Great Bay routinely experiences limited, short-lived ice cover within the bay. Consequently, the PGB population of spotted seals is compelled to breed at the coastal rookery of the Rimsky-Korsakov Archipelago (RKA) [3]. Currently, the archipelago is protected within the conservation area of the Far Eastern Marine Reserve. The PGB spotted seals can be distinguished from spotted seals of other populations by their larger body size, distinctive coloration of the newborn pelt, and several meristic characteristics [3,4]. The spotted seal population of PGB is the southernmost and least abundant in Russia. In the 1970s, it was assessed at just several hundred individuals [4], but over the past half century, it has grown to approximately 4000 individuals [5]. This increase has been attributed to protective measures applied by the Far Eastern Marine Reserve at the rookery of local spotted seals. The breeding season of the spotted seal in PGB is independent of ice conditions and is markedly prolonged, spanning from January to April [3,6]. At the end of spring and in early summer, once breeding and molting are complete, spotted seals leave PGB en masse and travel to summer–autumn feeding grounds in the north of the ES/SJ and the south of the OS [3,7]. Some spotted seals also travel southward along the Korean Peninsula. During the summer–autumn period, a portion of the population remains in PGB [8]. Nesterenko and Katin [8] estimated the number of spotted seals remaining in PGB during the summer at 450–500 individuals, which at that time accounted for 20% of the population. This remaining portion of the population has been termed “resident,” in contrast to the “migrating” seals that leave PGB [8]. It has been speculated that genetic isolation may exist between these groups [9]. Adult spotted seals tend to return to the same islands within RKA, and several individually recognizable (marked) seals have remained at the rookery across consecutive summer seasons [8]. The migratory routes and spatial characteristics of migrating spotted seals from PGB are strongly understudied.

The reasons for the co-occurrence of two opposite life history tactics within the PGB population of spotted seals are unclear. It is plausible to assume that juvenile spotted seals (<3 years old) can develop their migratory behavior in early stages of life, which makes them a particularly valuable subject for research of life history tactics. The migratory behavior of juvenile spotted seals warrants particular attention, as this group is the most vulnerable and least experienced in the local population. To date, only three juvenile seals from PGB have been tagged with satellite tags, and all undertook long foraging migrations [7]. The spatial characteristics of seals remaining in PGB during the summer–autumn season have not previously been assessed.

The primary aim of this research is to study the migratory behavior of juvenile spotted seals from the population of PGB. The objectives were to describe the utilization of space by juvenile spotted seals year-round; to confirm the emergence of different migratory tactics in juvenile spotted seals during the warm season; and to characterize these tactics by their spatial and speed-related properties.

## 2. Materials and Methods

**Figure 1:**
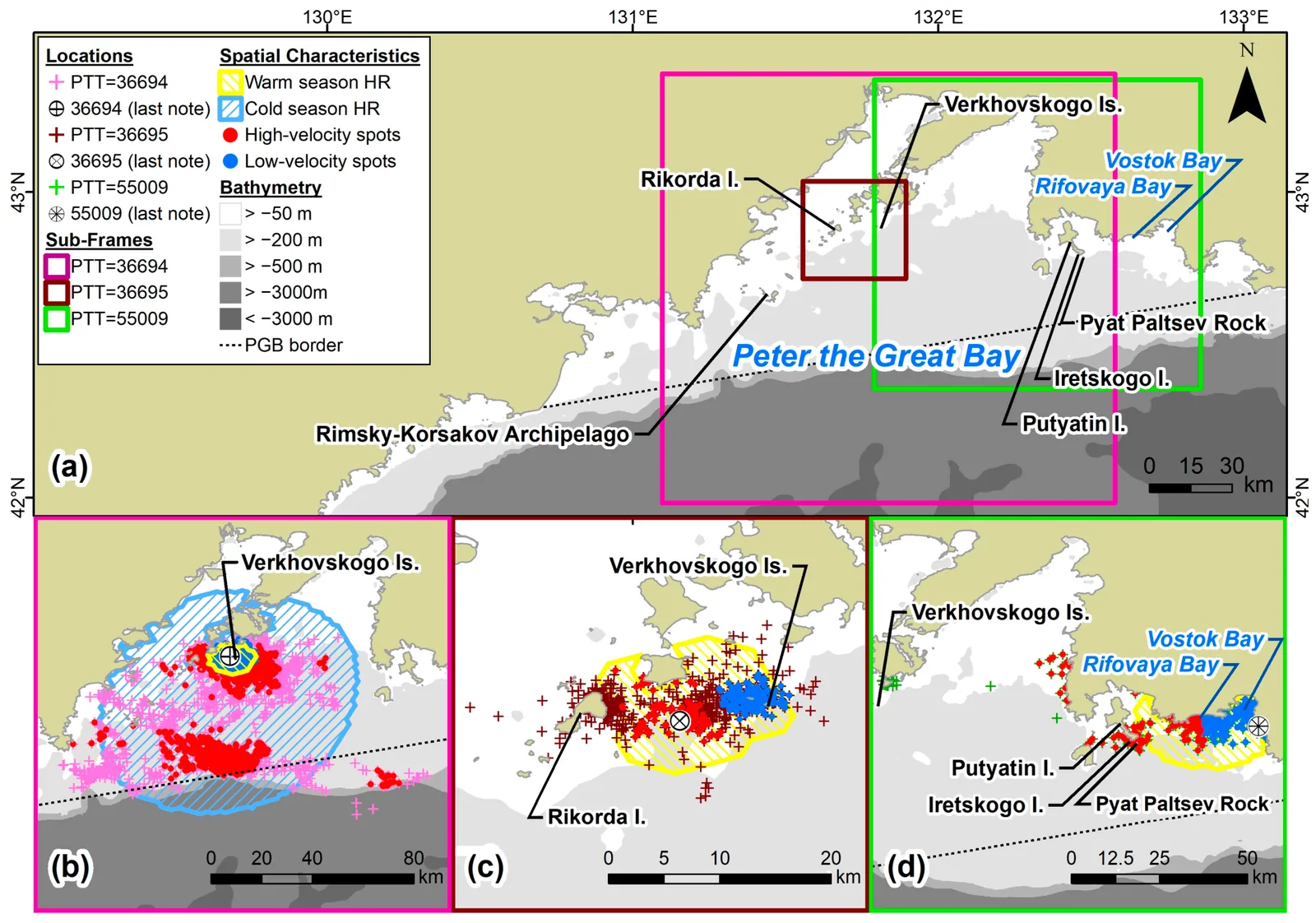
Spatial distribution of locations of spotted seals tagged at Verkhovskogo Island that remained in Peter the Great Bay: #36694 (**b**), #36695 (**c**), and #55009 (**d**); the topography of Bay shown in (**a**).

To assess the spatial characteristics of spotted seals from the PGB population, satellite tags were deployed on nine spotted seals between 2017 and 2022: five were weaned pups, three were juvenile seals aged between 1 and 2 years, and one was a 2-year-old subadult (Table 1). The age of all seals was determined by means of external signs, such as body mass, length, girth, and claw size. No body parts (e.g., teeth, claws) were collected from the seals to determine age under laboratory conditions. Tagging was conducted after the breeding season. Four seals were tagged at the rookery of RKA, while the remaining five were tagged at nearby haul-outs situated 53 km northeast of RKA (Verkhovskogo Islands; hereinafter: VI) (Figure 1). Satellite tags (SPOT-293A; ARGOS SPOT, Wildlife Computers, Inc., Redmond, WA, USA) were used. Tags were attached to the crown of each seal’s head using Loctite^TM^ Type 422 glue (Henkel Corp., Düsseldorf, Germany) or a two-component epoxy glue. Seals were released immediately after tagging. All spotted seals tagged in this study were born at the rookery on RKA, as no other breeding colonies of the species exist within PGB.

All received location data were preprocessed using the ARGOS Kalman filtering algorithm and subsequently underwent additional preprocessing with the SDA filtering algorithm [10], implemented in the “argosfilter 0.70” R package [11]. The threshold values used for the SDA parameters to remove records from the dataset were as follows: speeds exceeding 3.8 m/s over travel distances greater than 5 km [12] and angles between successive locations greater than 15° at distances exceeding 2.5 km and greater than 25° at distances exceeding 5 km. In addition, records indicating speeds greater than 10 m/s (regardless of travel distance) or locations situated more than 1 km inland from the shoreline were removed.

Satellite tags are typically shed during the annual molting of spotted seals; however, they may occasionally be lost earlier for various reasons. In such cases, tags lost within the tidal zone may continue transmitting location data because the wet/dry sensor can keep switching tags on and off in response to the diurnal tidal cycles. To identify and remove such records from the dataset, each tag was operated for 1 day in an open area at a site with precisely known coordinates. The coordinates were determined using a Garmin eTrex 30x GPS/GLONASS handheld navigation device. For each received record, the Euclidean distance (deviation) between the observed coordinates provided by ARGOS and the true coordinates measured using GPS/GLONASS was calculated. These deviations were compiled into a control set of biases. Subsequently, for each received field record, the deviation of the observed coordinates from a “moving” center of mass was calculated, with the center of mass defined as a 5-day moving average of the X and Y coordinates. These deviations were then grouped into 5-day sequences calculated using an analogous “moving” procedure and compared with the control set of bias using a *t*-test (*p* = 0.05). If no statistically significant difference was detected between the 5-day datasets of observed deviations and the control set of bias for 30 consecutive days, the tag was considered lost within the tidal zone.

Statistically significant areas used by individual spotted seals were identified using the hotspot analysis method [13]. This method proportionally compares densities of locations in aggregations of neighboring polygons (cells with side lengths of 5 km) to densities of locations in the bigger polygon that envelops a presumable range of PGB-spotted seals (i.e., ES/SJ, OS, and near-shore Japan). The disparity was considered significant when the positive score of the assigned Z(Gi*) statistic conformed to the 95% level of confidence (*p* = 0.05). Clusters of areas with high densities of locations were designated significant to spotted seals (hereinafter key areas). Within these key areas, spotted seals were observed significantly more often than expected relative to other parts of their range. This method showed limited use in application to the distribution of spotted seals that remained in PGB, due to the small sizes of their summer habitats.

The hotspot analysis method was also used to identify locations with statistically significant changes in the horizontal movement speed of spotted seals. In that case speeds in aggregations of neighboring locations were proportionally compared to speeds observed through whole tracks of individual spotted seals. Locations classified as “low-velocity spots” or “high-velocity spots” were marking statistically significant (*p* = 0.05) clusters of points with consistently decreased and elevated, respectively, horizontal movement speed. Repeated decreases in horizontal movement speed were interpreted as markers of potential foraging, resting, or social behavior, as seals may spend more time submerged during such periods. Large key areas with clusters of low-velocity spots were classified as primary resting and foraging grounds when individual spotted seals repeatedly used them for extended periods. All other key areas were classified as secondary.

To examine intraseasonal changes in the spatial distribution of spotted seals, their home ranges (hereinafter HRs) were delineated, defined as the areas within which individual spotted seals could be found with a 95% probability [14]. The warm season was defined as the period from May to October or, for migratory individuals, from the completion of summer migration (typically late June to early August [7]) to October. The cold season was defined as beginning in November or, for migratory individuals, after the completion of the return autumn migration (typically occurring between November and December [7]). Kernel density estimation was used to smooth the probability density distribution, with a raster cell size of 1500 m. This method does not allow statistically meaningful conclusions about areas used by spotted seals but can be used to compare changes in utilization of individual areas over time.

A direct comparison of movement speeds among spotted seals could be misleading, as migratory individuals would be expected to exhibit higher average movement speeds because they spend a greater proportion of their time traveling. To address this issue, only the speeds observed during transit passages were compared. To identify the transit movements, the tracks of individual spotted seals were divided into sequences of 8-h intervals, and the Rayleigh test for uniformity [15] was applied at each interval. The Rayleigh test assesses departures from a uniform circular distribution and therefore can be used to determine whether a consistent travel direction is present within a given 8-h interval. When a significant average travel direction was detected, all records within a tested 8-h period were classified as part of a transit movement. The probability associated with the Rayleigh statistic (R) was estimated using its exponential approximation. Subsequently, the spotted seals were assigned to one of two clusters using the k-means clustering method [16]. In k-means clustering the data are iteratively allocated to different clusters, each characterized by its own center of mass (centroid). After each reallocation, centroids are reevaluated. The process of reallocation aims to achieve a configuration of clusters at which every datum belongs to a cluster with a centroid closest to it. The process was ceased when no new changes in the configuration of clusters resulted in a decrease in the adopted proximity metric (*dE*; the Euclidean distance). Clustering was based on the median movement speed of each individual during transit movements.

To compare differences between datasets, permutation tests were used [17]. The function used to construct the achieved significance level (*ASL*, permutation test statistic) was comparing a difference between means of tested variables. During each replication of the test, the difference of means from the observed dataset (*Θ*) was fixed; at the same time, the difference of means from the permuted dataset (Θ*) was a result of random re-sampling without replacement from the same dataset. In sequence of replications, whenever Θ* was bigger than *Θ*, *ASL* was increased by 0.001. All permutation tests were run through 1000 replications. The critical value for the permutation test statistic was set at *ASL* = 0.05 (which is analogous to *p* = 0.05). The variables subjected to permutation tests are separately specified under lower sections.

All calculations were performed using the Asia North Equidistant Conic projection, whereas figures and other illustrative materials were prepared in WGS 84.

After preprocessing, 23,582 records remained in the working dataset (Table 1). During the study, open-source map data from OpenStreetMap [18] were used for shoreline information and data from the on-line resource “GEBCO Maps” [19] for bathymetry. All data were processed using ArcMap 10.8, Microsoft Excel, R version 3.4.3, and GraphPad Prism 6.

## 3. Results

### 3.1. Migration of Seals from the Verkhovskogo Islands

Five spotted seals, ranging from weaned pups to subadults, were tagged between 24 May and 7 June 2019 at the VI haul-out. The deployed tags remained functional for 21–276 days (Table 1). Spotted seals #36694, #36695, and #55009 generally remained within the boundaries of PGB (Figure 1b,c) (hereinafter “nonmigrating”). The at-sea movements of female #36694 were followed throughout the winter and into the following spring. From June to October, this female occupied a limited area of the sea. In November, #36694 began to undertake passages into the seaward part of PGB, toward the area of the depth drop-off beyond the 200-m isobath (Figure 1b). The HR of this female was S_HR_ = 105 km^2^ during the warm season and S_HR_ = 4336 km^2^ during the cold season (Table 2).

The spatial characteristics of male #36695 and female #55009 were examined only during the warm season. Both spotted seals spent the summer in compact areas. Male #36695 generally inhabited the water area spanning between VI and Rikorda Island (S_HR_ = 88 km^2^; Figure 1c). After its tag ceased transmitting on 14 July 2017, male #36695 was opportunistically resighted by recreational divers on 29 September 2019, at VI [20]. The seal was photo-identified by the spot pattern on its pelt. By the end of May 2019, female #55009 had moved to Rifovaya Bay and remained there until the end of August (S_HR_ = 312 km^2^; Figure 1d). The female regularly visited nearby Vostok Bay and the southeastern end of Putyatin Island, presumably to attend the haul-outs situated at Pyat Paltsev Rock and Iretskogo Island. The tag of #55009 functioned smoothly until 30 August 2019, but then ceased transmitting and was reactivated on 10 October 2019. The tag continued to function until March 2020; however, during the remaining phase of its operation, the deviations in the location of the tag from the 5-day “moving” center of mass did not exceed the control set of measured bias (*p* < 0.05). Data after August 30 were, therefore, removed from the dataset.

**Figure 2:**
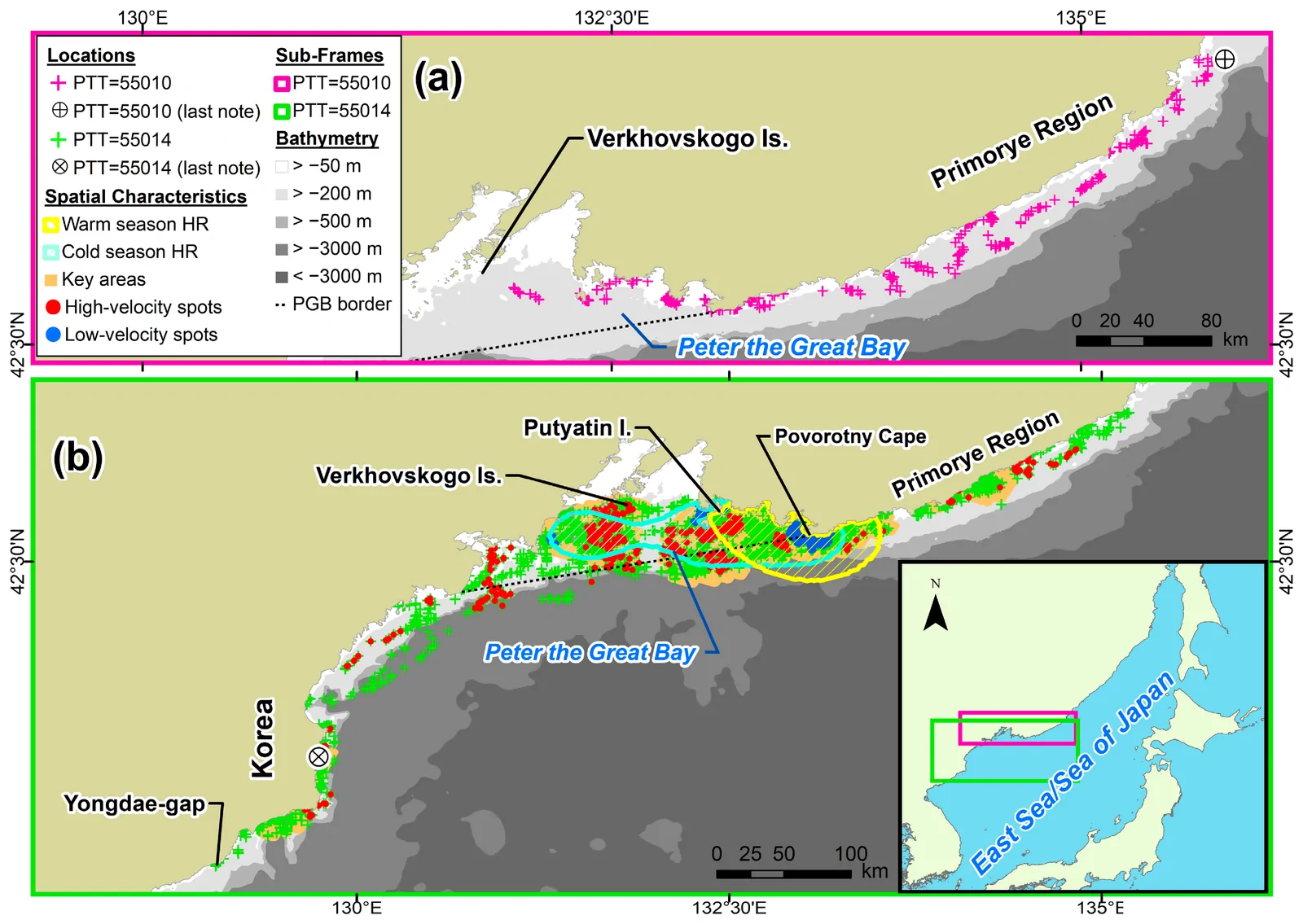
Spatial distribution of spotted seals tagged at Verkhovskogo Island that left Peter the Great Bay: (**a**) #55010 and (**b**) #55014.

The remaining spotted seals from the group tagged at VI (#55010 and #55014) left PGB and moved within the shelf area (Figure 2). Female #55010 left PGB on 19 June 2019 and traveled eastward along the coast of the Primorye Region (Figure 2a). The spatial characteristics of this spotted seal were not assessed due to insufficient data. The last transmission from #55010 was received in late June, suggesting that the female may have initiated a foraging migration; however, it could not be tracked further due to the short service time of the tag.

The yearling female #55014 left PGB in June 2019 and traveled more than 270 km in a northeastward direction (Figure 2b). She then abruptly stopped moving and returned to PGB. Until January 2020, she remained within the main key area adjacent to Povorotny Cape (the easternmost point of PGB). During the warm season, the HR of female #55014 was S_HR_ = 2986 km^2^ (Table 2). The seal’s primary feeding ground was situated around Povorotny Cape and to the west of Putyatin Island (Figure 2b; see Key areas and low-velocity spots). After November, the HR of this spotted seal began to grow, and by January 2020, it had increased to S_HR_ = 4460 km^2^ (Table 2). In early 2020, the female left PGB again and moved southwestward until she reached Yongdae-gap, 340 km from the tagging spot (Figure 2b). While moving along the coast of Korea, female #55014 formed several secondary key areas in which she did not slow her pace to a statistically significant degree. The tag ceased transmitting on 24 February 2020.

### 3.2. Migration of Seals from the Rimskiy-Korsakov Archipelago

**Figure 3:**
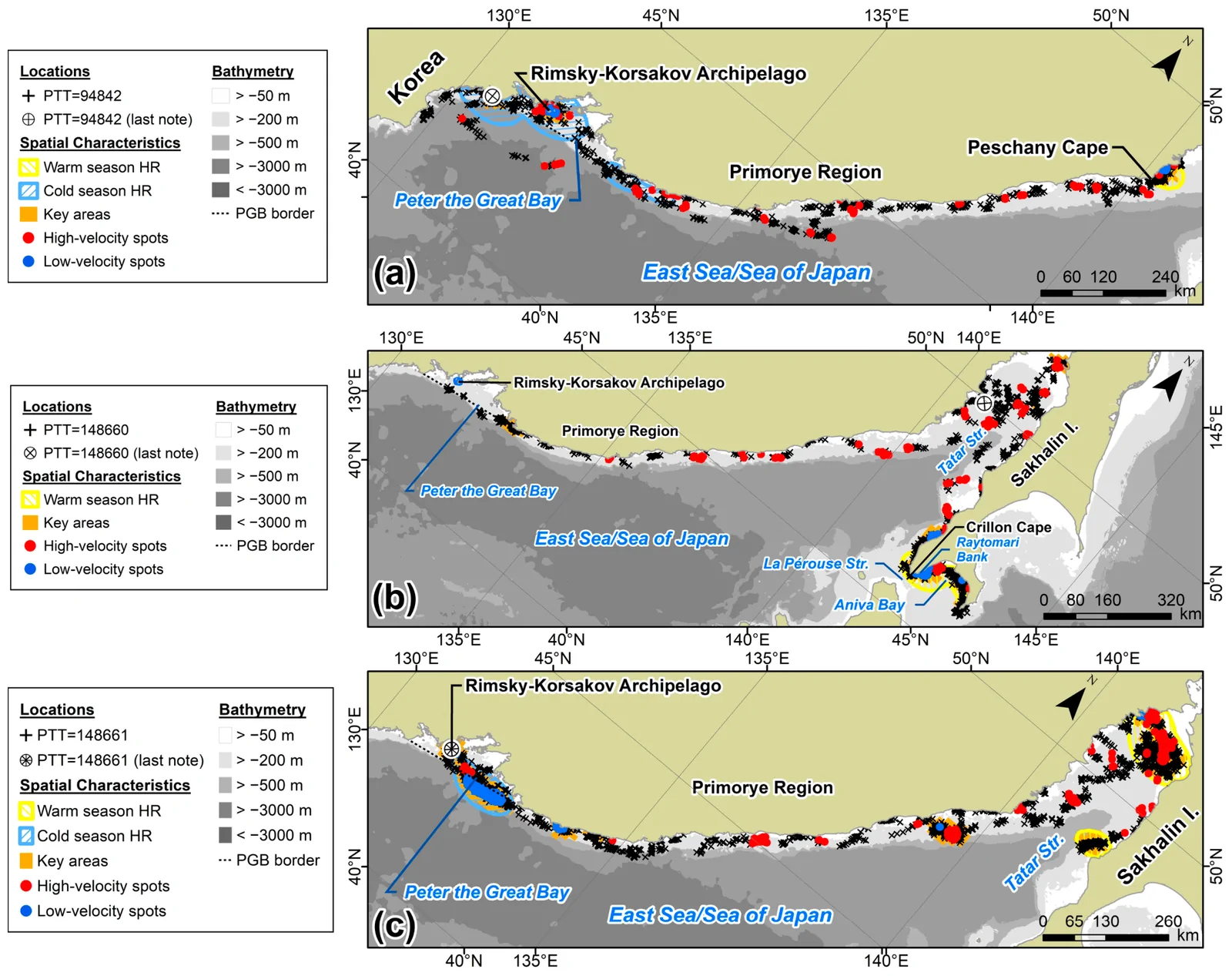
Spatial distribution of spotted seals tagged at the Peter the Great Bay rookery in 2017: (**a**) #94842, (**b**) #148660 and (**c**) #148661. Adopted with permission from Trukhin et al. [7].

Information on the spatial and speed characteristics of three juvenile spotted seals tagged in 2017 (Table 1) was partially used in this analysis and has been published previously [7]. All three seals undertook long-distance foraging migrations to the north of the ES/SJ and to the south of the OS during the warm season (Figure 3). Female #94842 spent the warm season in a compact area north of Peschany Cape (Figure 3a); male #14660 summered in Aniva Bay, mostly over the Raytomari Bank (Figure 3b); and male #14661 foraged in the northern part of the Tatar Strait (Figure 3c). Two seals (#94842 and #14661) were observed returning to PGB and spending the winter there and in nearby coastal areas of Korea [7]. The spotted seals had large HRs during both the warm and cold seasons (Table 2).

**Figure 4:**
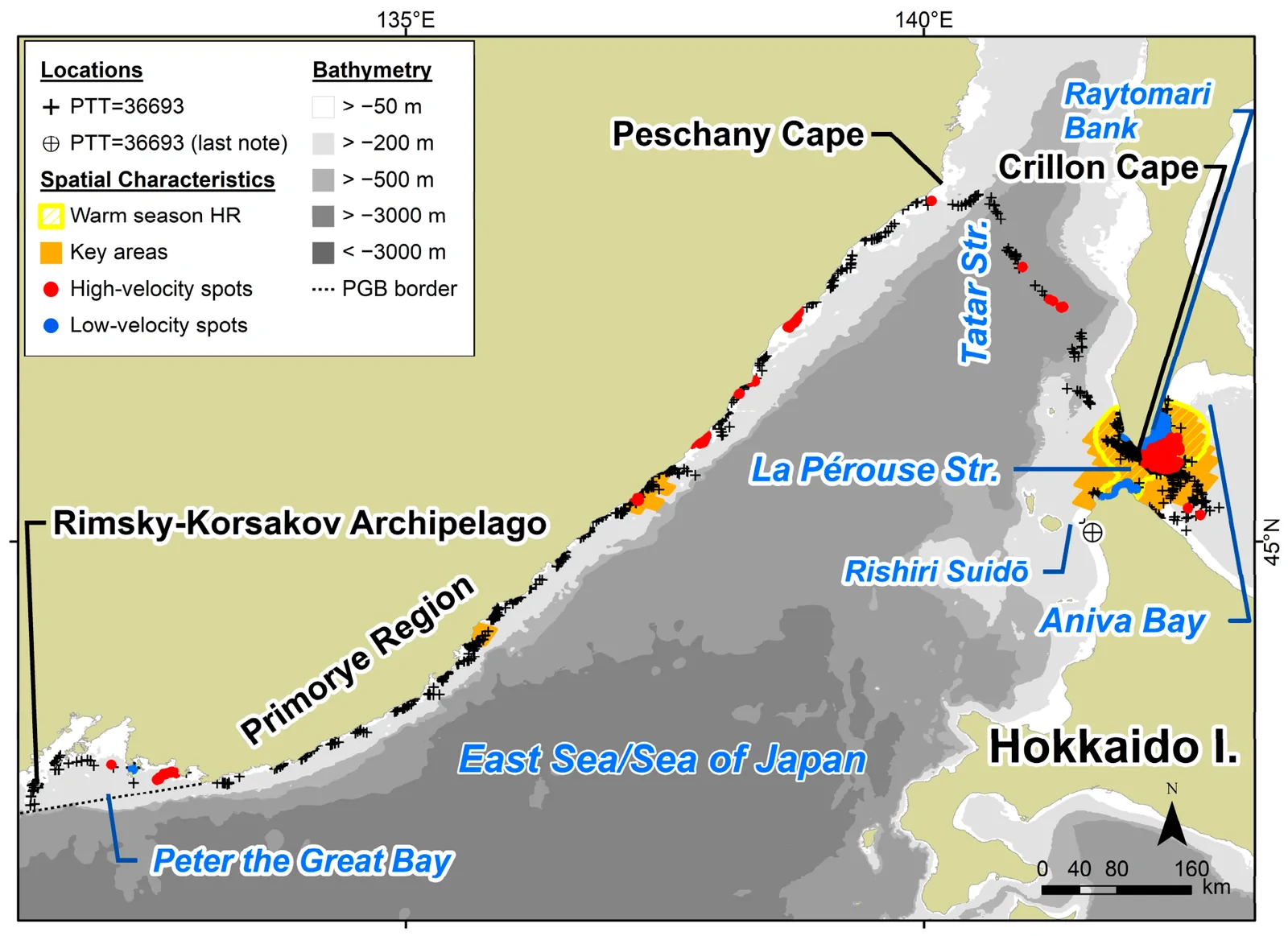
Spatial distribution of juvenile spotted seal #36693 tagged at the Peter the Great Bay rookery.

The under-yearling female #36693 was tagged at RKA on 25 May 2022 (Table 1). The female left PGB 3 days later and traveled along the ES/SJ coast toward the Tatar Strait (Figure 4). On 19–22 June, the female crossed the Tatar Strait between Peschany Cape and Crillon Cape, and on 23 June, she entered her main foraging area, which extended across the waters of the ES/JS and the OS on both sides of La Pérouse Strait (see Figure 4, Key areas). Female #36693 spent most of her foraging time at Rayatomari Bank and south of Crillon Cape, where she repeatedly moved between Sakhalin Island and Hokkaido Island and back, along the local belt of cold waters that emerges annually in summer along the front of Soya Warm Current [21,22,23]. During the warm season, the HR of this spotted seal was S_HR_ = 4447 km^2^ (Table 2). The seal died in a fishing net in Rishiri Suidō on 5 October 2022 [24].

### 3.3. Spatial and Speed Characteristics of the Peter the Great Bay Spotted Seals

The proportions of transit traveling records within the activity budgets of spotted seals tagged at different sites partially overlapped (2–25% transit records in spotted seals tagged at VI vs. 15–21% in those tagged at RKA; Table 3). The medians did not differ statistically between tagging locations (permutation test, ASL > 0.05). The median transit speeds were 0.50–0.88 m/s and 0.93–1.03 m/s, respectively. Median speeds of seals tagged at VI and PGB were significantly different (ASL < 0.05), which was the basis for assigning the spotted seals into two distinct groups (clusters). When all spotted seals were assigned to clusters based on their median speeds, the intragroup centroids were 0.59 m/s for the first k-cluster and 0.94 m/s for the second; the proximity function was dE = 0.20 m/s. After pooling all locations by k-cluster, the median transit speed of seals within the first k-cluster was Me = 0.42 m/s (interquartile range [IQR] = 0.16–0.99 m/s), and Me = 0.62 m/s (IQR = 0.25–1.38 m/s) within the second k-cluster. The difference between median speeds on transit passages pooled by k-clusters was statistically significant (ASL < 0.05). The first k-cluster consisted of spotted seals that did not leave PGB (nonmigrating spotted seals #55009, #36694, and #36695), while the second k-cluster included all other spotted seals (hereinafter, migrating spotted seals; note that this group includes at least one formally nonmigrating individual, #55014) (Table 3).

The spotted seals tagged at the VI haul-out were distributed across k-clusters, indicating that tagging location did not influence the seals’ tendencies to use high or low traveling speeds (Fisher’s exact test, *p* = 0.12). Likewise, age had no apparent effect: grouping by age (“under-yearlings” vs. “older than 1 year”) was not statistically significant between k-clusters (Fisher’s exact test, *p* = 0.36). The robustness of these findings can be limited due to the small sample size in this study.

Migrating spotted seals exhibited generally higher levels of activity: in addition to higher traveling speeds, they had a greater proportion of transit records (on average, 19% vs. 7% in nonmigrating spotted seals; Table 3). They also used larger sea areas during the warm season: on average, nonmigrating spotted seals had HRs of only S_HR_ = 168 km^2^ (95% confidence interval [CI] = 272 km^2^), whereas migrating spotted seals used HR ranges tens of times larger (S_HR_ = 4754 km^2^, 95% CI = 4260 km^2^; Table 2). Warm-season water areas differed significantly between seals in the two k-clusters (ASL < 0.05).

Movements of one nonmigrating (#36694) and three migrating (#94842, #148661, and #55014) spotted seals were tracked through the cold season (Table 2). During the cold season, the sizes of the sea areas used by spotted seals were comparable between k-clusters (S_HR_ = 4336 km^2^ and S_HR_ = 7120 km^2^, respectively) and did not differ statistically (ASL > 0.05). This pattern differed from that observed during the warm season. This change occurred because, in late autumn, the HRs of nonmigrating spotted seals increased significantly—from several hundred to several thousand square kilometers (e.g., Figure 1b). At the same time, migrating seals tended to use large sea areas regardless of season (Table 2).

## 4. Discussion

This study corroborates earlier findings of a migratory route from PGB to foraging grounds along the northern coast of the ES/SJ [7,25,26]. Four of the nine tagged seals reliably spent the warm season within the Tatar Strait and in the southern part of the OS (Figure 3 and Figure 4). In addition, one yearling female (#55010; Figure 2a) tagged at VI presumably began her summer migration in a northeastward direction, but her tag then ceased transmitting, and the female was lost from tracking. The presence of a migrating individual among the juvenile seals at the peripheral haul-out of VI is consistent with our earlier finding that, before the onset of long migrations, juvenile spotted seals may spend time in small areas situated close to the border of larger key areas they are about to leave [7]. This behavior may be part of the preparation for a long migration.

The juvenile tagged seals occasionally traveled long distances southward along the near-shore waters of Korea (see Figure 2b and Figure 3a). These travels did not qualify as foraging migrations, as the juvenile seals did not form the main key areas during them.

The tagging location seemingly did not affect seals’ tendency to use high or low travel speeds. A mixed group of spotted seals, tagged at both RKA and VI, was assigned to the high-speed k-cluster. Seals in that group exhibited 48% higher median transit speed compared with spotted seals in the low-speed k-cluster, which was composed exclusively of nonmigrating spotted seals, all tagged at VI (Table 3).

The observed difference in median speeds between the two k-clusters has high biological importance. Migrating spotted seals exhibited much higher activity levels during their transit passages, which should translate into substantially greater energy expenditure compared with nonmigrating seals. The proportion of transit records in the activity budget (Table 3) and the size of HRs during the warm season were also higher in migrating spotted seals (Table 2). It should be noted that the use of small water areas by juvenile spotted seals may be typical for nonmigrating individuals, as the same pattern has been observed in another spotted seal population [27].

The reasons for the dissimilarity in spatial and speed characteristics between migrating and nonmigrating spotted seals are not completely clear. Reports of repeated observations of the same individually recognizable spotted seals in PGB during summer, year after year [8], suggest a certain interannual stability within the nonmigrating group. Recent research on the genetics of PGB spotted seals revealed at least two mitochondrial lineages, leading Podlesnykh and Katin [9] to suggest genetic isolation of the PGB spotted seal population. If such isolation does exist, it could have occurred historically due to sea-level changes associated with Pleistocene or later glaciations. This hypothesis currently lacks sufficient corroboration from genetic data and requires a thorough evaluation of both migrating and nonmigrating spotted seals within the PGB population. This evaluation would necessitate sampling (skin biopsy and hair collection) of individual spotted seals whose affiliation with one or the other ecological group is unambiguous. Correctly attributing spotted seals to specific ecological groups will require a well-designed seal-monitoring program within PGB.

If it is assumed that nonmigrating spotted seals belong to a stable demographic unit within the PGB population, then their group might be expected to gain a competitive advantage in the form of lower energetic expenditure, which could result in their numerical dominance over migrating spotted seals within a conceivable timeframe [28]. However, as only about one-fifth of the population remains in PGB during the summer [8], the suggestion of competitive superiority of the nonmigrating group is questionable. It seems more plausible that the nonmigrating group emerges annually in response to multiple factors. A reasonable explanation for the low number of spotted seals remaining in PGB in summer may be the relatively lower availability or abundance of local nutrient resources. For example, in summer, some typically cold-water prey species that form the base of the spotted seals’ diet—such as walleye pollack (*Gadus chalcogrammus*) and navaga cod (*Eleginus gracilis*)—tend to migrate below the 50-m isobath [29], which can temporarily reduce their abundance in the shallow water areas of PGB (Figure 1a). To compensate for this summer-time decrease in prey availability, spotted seals in PGB may be forced to resort to trophic specialization, relying more heavily on cephalopods, such as Japanese common squid (*Todarodes pacificus*) [8]. Since the early 1990s, the Japanese common squid has dominated the nekton biomass in the ES/SJ [30], but its annual runs into PGB can be highly inconsistent, depending on short-term oceanographic conditions [31]. This makes it an unreliable nutrient source for the PGB spotted seal population. It is possible that the nonmigrating juvenile spotted seals can rely on local concentrations of nutrient resources that can be formed by relatively small, slow-pacing prey species such as humpy shrimp (*Pandalus goniurus*) and sculpins (*Cottidae gen.* sp.) [3]. Small patches of locally abundant biotopes that exist in proximity to traditional haul-outs of spotted seals can allow nonmigrating juvenile seals to remain in PGB during the warm season, while exhibiting low levels of activity. At the same time, when most juvenile seals face a broad situation of less predictable and available or exhaustible nutrient resources within PGB in the summer, they can travel to the northeast of ES/SJ. As during the warm season the cold-water prey species can be more available in colder areas, the migrating juvenile seals in the Tatar Strait and the OS can be less restricted by their energetic expenditures and possibly can keep a more active way of life, traveling faster or foraging in bigger HRs. This hypothesis is preliminary, and it needs further corroboration through research using bigger sample size data that should be supported by bio-logging techniques, such as stable isotope analysis of samples obtained from individually recognizable—preferably branded or plastic-tag marked—spotted seals, whose spatial characteristics are assessed throughout the warm season. It also requires dedicated comparative research of seasonal bio-productivity in PGB and in the areas used for warm-season foraging by migrating spotted seals.

Notably, some spotted seals may exhibit peculiar “hybrid” behavior. Female #55014 would formally be considered nonmigrating, as she did not perform (or possibly ceased) a foraging migration and never formed main key areas outside of PGB (Figure 2b). At the same time, she maintained high transit speeds and used large water areas during the summer. Also, the female #94842 maintained high transit speeds but spent her warm season within a compact area north of Peschany Cape (Figure 3a).

A single migrating female spotted seal (#36693) died in a human-related accident during her foraging trip along the northwestern coast of Hokkaido Island. The circumstances of the incident remain unclear, but the event highlights that conflict with fisheries represents a serious threat to juvenile spotted seals from the PGB population. Specialized research is needed to assess areas critical to PGB spotted seals in the nearshore waters of southwestern Sakhalin Island and northern Hokkaido Island and to determine whether migratory corridors (e.g., the Tatar Strait and the La Pérouse Strait) should be incorporated into conservation networks. At present, the breeding colony of spotted seals within PGB is adequately protected by the conservation area of the Far Eastern Marine Reserve, as no rookeries of this species exist beyond its protected boundary. Nevertheless, over time, the observed increase in the local spotted seal population [5] could lead to the establishment of new breeding colonies in PGB and will require additional protective measures to preserve the local population.

## 5. Conclusions

This study focused on spatial utilization plasticity during the juvenile stage of spotted seals from PGB (ES/SJ). Using satellite tag tracking, we confirmed that during the warm season, the PGB population can divide into two groups of seals with different behaviors. In total, nine juvenile seals were tagged during 2017–2022 in two locations within the bay: the RKA (main rookery) and the VI (nonbreeding haul-outs). One group of juvenile spotted seals left the bay on summer foraging migrations, traveling far to the northeast as far as the Tatar Strait and the OS, and returned in late autumn. The second group remained in the bay all year round. The seals in these two groups exhibit different speeds during transit travel. A relatively slow group of spotted seals remained in PGB. In addition to slower movements, this group conducted fewer transit passages and used smaller water areas. Migrating spotted seals have higher travel speeds and larger HR sizes. The small sample size in this study inevitably limits the robustness of the findings; nevertheless, this study is the first to show that seals born in the same location can exhibit markedly different spatial utilization. The underlying causes of this segregation remain unknown and represent a promising avenue for future research.

## Figures and Tables

**Table 1:** Information on tagged spotted seals and the working time of each tag.

Releasing Date	PTT	Working Time Of A Tag, Days	Tagging Location ^1^	Sex of Seal	Body Mass, kg	Age of Seal	Number of Datums Accepted for Analysis
18 May 2017	#94842 ^2^	316	RKA	F	28.4	pup	2780
18 May 2017	#148660 ^2^	207	RKA	M	35.6	yearling	2154
19 May 2017	#148661 ^2^	351	RKA	M	32.2	pup	3146
24 May 2019	#55009	98	VI	F	26.2	pup	1944
1 June 2019	#36695	43	VI	M	>50	2-years old	761
3 June 2019	#36694	276	VI	F	28.0	yearling	4602
4 June 2019	#55014	265	VI	F	25.2	yearling	4827
7 June 2019	#55010	21	VI	F	24.2	pup	440
25 May 2022	#36693	133	RKA	F	27.1	pup	2928

^1^ RKA, Rimsky-Korsakov Archipelago; VI, Verkhovskogo Island. ^2^ Data were adopted from Trukhin et al. [7], except for the number of datums accepted for analysis.

**Table 2:** Home range areas for spotted seals from different k-clusters.

PTT	Season	k-Cluster ^1^	HR Area (km^2^)	Average Area Per k-Cluster ^2^, (km^2^)
#36694	warm	1	105	168
#36695	warm	1	88	
#55009	warm	1	312	
#148660	warm	2	5472	4754
#148661	warm	2	9638	
#36693	warm	2	4447	
#55014	warm	2	2986	
#94842	warm	2	1229	
#36694	cold	1	4336	4336
#148661	cold	2	5780	7120
#55014	cold	2	4460	
#94842	cold	2	11,121	

^1^ k-cluster includes nonmigrating (1) and migrating (2) spotted seals. ^2^ Averages were calculated across seasons and k-clusters.

**Table 3:** Grouping of spotted seals into k-clusters based on median transit speed.

PTT	Tagging Location	Proportion of Transit Traveling Records (%)	Median Transit Speed (m/s)	k-Cluster ^1^
#94842	RKA	15%	0.96	2
#148660	RKA	21%	0.93	2
#148661	RKA	19%	1.01	2
#55009	VI	2%	0.65	1
#36695	VI	9%	0.5	1
#36694	VI	10%	0.62	1
#55014	VI	16%	0.88	2
#55010	VI	25%	0.82	2
#36693	RKA	18%	1.03	2

^1^ k-cluster includes nonmigrating (1) and migrating (2) spotted seals.

## Data Availability

The original data are available at Mendeley Data: Ryazanov S.; Permyakov P.; Trukhin A.; Lobanov V.; Kim H.W.; Kim S. Satellite tracking data of young spotted seals (*Phoca largha*) from Peter the Great Bay, 2017–2022. Mendeley Data 2026, Version 1, doi: 10.17632/kpswgmnxhy.1.

## Abbreviations

ES/SJ	the East Sea/Sea of Japan
OS	the Okhotsk Sea
PGB	Peter the Great Bay
RKA	Rimsky-Korsakov Archipelago
VI	Verkhovskogo Islands
HR	home range
S_HR_	area of home range
ASL	achieved significance level
Me	median
IQR	interquartile range
